# Precancerous lesions of the cervix and its determinants among Ethiopian women: Systematic review and meta-analysis

**DOI:** 10.1371/journal.pone.0240353

**Published:** 2020-10-28

**Authors:** Binalfew Tsehay, Mekbeb Afework

**Affiliations:** 1 Department of Biomedical Sciences, Debre Markos University, Debre Markos, Ethiopia; 2 Department of Anatomy, Addis Ababa University, Addis Ababa, Ethiopia; Istituto Nazionale Tumori IRCCS Fondazione Pascale, ITALY

## Abstract

**Introduction:**

Precancerous lesions of the cervix are changes in cervical cells that make them more likely to develop into cancer. Understanding the prevalence and determinants of the precancerous lesions of the cervix among women helps to take an action like vaccination programs, improving screening coverage, and close management and follow-up which could decrease the morbidity and mortality caused by cervical cancer.

**Materials and methods:**

The international databases, PubMed/MEDLINE, Web of Science, EMBASE, CINAHL, Google Scholar, Science Direct and Cochrane Library and unpublished reports were systematically searched. Two authors independently extracted all necessary data using a standardized data extraction format. STATA 14 statistical software was employed to analyse the data. The Cochrane Q test statistics and I^2^ tests were used to assess the heterogeneity between the studies. A random-effect model was computed to estimate the pooled prevalence of precancerous lesions of the cervix in Ethiopia. Determinants of the precancerous lesion of cervix (early initiation of sexual intercourse, multiple sexual partners, and history of sexually transmitted infection) were analysed.

**Results:**

Thirteen studies fulfilled the inclusion criteria and included in the meta-analysis. The I^2^ test result showed high heterogeneity (I2, 93.2%, p = <0.001). Using the random effect analysis, the pooled prevalence of precancerous lesions of the cervix among women in Ethiopia was 14.21% (95% CI (10.49, 17.94). After adjusting of publication bias using trim-and-fill method, the pooled prevalence was 9.43% (95% CI (5.23, 13.62). Women who had multiple sexual partners in their lifetime (OR:2.67 CI: 1.49,4.79) and had a history of sexually transmitted infections (OR:6.22 CI: 2.99,12.92) were more likely to have a precancerous lesion of the cervix.

**Conclusion:**

The pooled prevalence of the precancerous lesions of the cervix among Ethiopian women was 9.43%. It was associated with having multiple sexual partners and a history of sexually transmitted infections.

## Introduction

Precancerous lesions of the cervix are changes to the cervical cells in an area called the transformation zone. According to the World Health Organization (WHO), this change can exist at any of three stages: cervical intraepithelial neoplasia stage 1 (CIN1), stage 2(CIN2), or stage 3 (CIN3). These conditions are not yet cancer. But if they aren’t treated, CIN2 or CIN3 collectively referred to as cervical intraepithelial neoplasia stage 2 plus (CIN2+) can progress to cervical cancer [1, 2].

Cervical cancer is the 3^rd^ most common cancer among women worldwide, with an estimated 569,847 new cases and 311,365 deaths in 2018 [3]. The burden of cervical cancer is high in Central and South America, East Africa, South and-South-East Asia, the Western Pacific, and mainly worse in sub- Saharan Africa [4]. In Ethiopia, Cervical cancer is the 2^nd^ most common female cancer in women aged 15 to 44 years. About 6,294 new cervical cancer cases are diagnosed annually [5]. Histologically most cases are squamous cell carcinoma followed by adenocarcinoma [6, 7].

The aim of cervical cancer screening is to identify women at risk and to ensure appropriate follow-up for those who have a positive or an abnormal test result. World health organization recommends ‘screen-and-treat’ approach for screening and treatment of precancerous lesions for cervical cancer prevention. Treatment is provided soon or, ideally, immediately after a positive screening test. Screening tests include a human papillomavirus (HPV) test, visual inspection with acetic acid (VIA), and cytology (Pap test) [2].

About 70% of women in the United States and Europe are screened at least once, every five years. However, in developing countries, only 5% of women are screened in this same time period [8].

Early detection and management of precancerous lesions of cervix require policymakers and technical input. Challenges faced by countries are lack of awareness of people about cervical cancer, absence of a policy framework, inadequate infrastructure, insufficient data, and evidence [3, 9].

Understanding the prevalence and determinants of the precancerous lesions of the cervix among women helps to take actions like vaccination programs, improving screening coverage, and close management and follow-up which could decrease the morbidity and mortality caused by cervical cancer.

In Ethiopia, different studies were conducted to determine the prevalence and factors associated with precancerous lesions of the cervix. These studies reported that the prevalence of precancerous lesions among women ranges from 6.7% to 27.7% [10–21]. The common factors reported by the studies were early initiations of first sexual intercourse, multiple sexual partners, and a history of STI [11, 12, 14, 16, 17, 19–23].

The findings from these studies were highly variable and inconsistent. Therefore, the main aim of this systematic review and meta-analysis is to determine the pooled prevalence and determinants of precancerous lesions of the cervix among women in Ethiopia using available studies. The findings of this review will have an input to policymakers and program planners in the design of appropriate interventions to decrease cervical cancer in the country. In addition, the review may serve as a baseline for future research in related topics.

## Materials and methods

### Study design and setting

A systematic review and meta-analysis was conducted to estimate the pooled prevalence and determinants of precancerous lesions of the cervix among Ethiopian women.

### Search strategies

This meta-analysis was conducted according to the Preferred Reporting Items for Systematic Reviews and Meta-Analysis (PRISMA) [24] (S1 Table). A comprehensive search with no date limits and written in the English language was performed to access relevant articles in the following databases: PubMed/MEDLINE, Google Scholar, Web of Science, EMBASE, CINAHL, Science Direct, and Cochrane Library. In addition, observational studies were searched through the review of reference lists and journals like PLOS one, BMC. Unpublished papers were searched through university databases. Studies identified by our search strategy were retrieved and managed using Endnote X7 (Thomson Reuters, Philadelphia, PA, USA) software. This comprehensive search of the literature was conducted between the 1^st^ of October to the 1^st^ of November, 2019 (S1 File).

### Eligibility criteria

#### Inclusion criteria

Study area: Only studies conducted in Ethiopia.

Population: Studies involving women eligible for screening services.

Publication condition: Both published and unpublished articles were included.

Study design: All observational study designs (i.e., Cross-sectional and/ or case-control) reporting the prevalence of precancerous lesion of the cervix and/ or factor were eligible for this review.

Language: Only articles reported in the English language were considered.

#### Exclusion criteria

Articles regarding already confirmed cancerous lesions; Records with unrelated outcome measures; and articles with missing or insufficient outcomes; Reviews, commentaries, editorial, case series/report, and patient stories were excluded from the systematic review.

### Measurement of outcome variables

This study has two main outcomes. The first outcome was the pooled prevalence of the precancerous lesions of the cervix among women in Ethiopia, estimated as the total number of women with lesion divided by the total number of women taking part in the study multiplied by 100. The second outcome of this study was to identify the determinants of precancerous lesions of the cervix among Ethiopian women. For the second outcome, we assessed the association between precancerous lesions of the cervix and its determinants in the form of the log odds ratio. For major determinants, the odds ratio was calculated based on binary outcomes from the primary studies.

### Quality assessment

Quality assessment was performed using the 22-items “Strengthening the Reporting of Observational Studies in Epidemiology” (STROBE) checklist that relates to the title, abstract, introduction, methods, results, and discussion sections of articles [25]. Studies that scored more than 75% (17 out of the 22 items) were considered to have good quality (S2 Table).

### Data extraction and recording

The data from the selected articles were extracted using a standardized data extraction format, adapted from the Joanna Briggs Institute (JBI), independently by two authors (BT and MA). Any disagreements during the data extraction were resolved through discussion and consensus. For the first outcome (prevalence), the data extraction format included the author, publication year, region(s) of the country where the study was conducted, sample size, study population, and prevalence with 95% CI. For the second outcome (determinants), data were extracted in a format of two-by-two tables, and then the log odds ratio for early initiations of first sexual intercourse, multiple sexual partners, and history of an STI was calculated based on the findings of the original studies.

### Data analysis

The extracted data were entered into an excel sheet and imported to STATA version 14 for analysis. Heterogeneity among reported prevalence was assessed by using the inverse variance (I2) with Cochran Q statistics of 25, 50 and 75% as low, moderate and severe heterogeneity respectively with a p-value less than 0.05 [26]. In this meta-analysis, I^2^ values were found to be high (> 75%). Since this value is a definitive indicator of significant-high heterogeneity, the random-effects meta-analysis model was used to estimate the pooled prevalence of precancerous lesions of the cervix. The funnel plot was also used to visualize the presence of heterogeneity graphically. Possible differences between the studies were explored by subgroup analyses. The finding was presented using a forest plot with respective odds ratios and 95% confidence intervals. Evidence of publication bias was assessed using both Egger’s, and Begg’s tests with a p-value of less than 0.05 as a cut-off point [27, 28]. For the second outcomes, pooled odds ratios with 95% CI for each factor were used to determine the association between precancerous lesions of the cervix and its factors (early initiations of first sexual intercourse, multiple sexual partners and history of STI).

## Results

### Selection of studies

**Fig 1:**
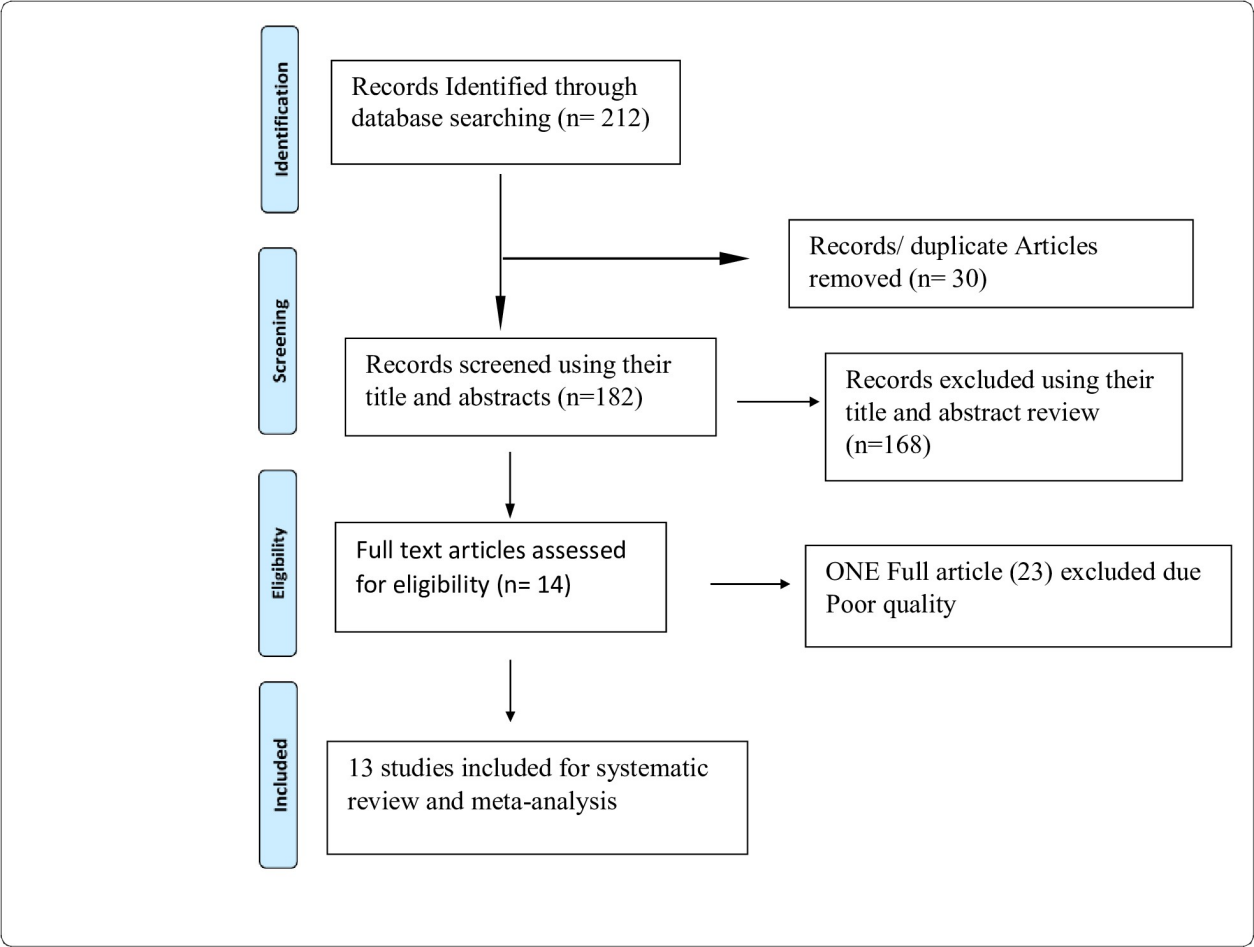
Flow diagram of the studies included in the review.

We searched 212 articles through the electronic searches, of which we excluded 168 articles based on the title. From the remaining 44 articles, 30 duplicate articles were excluded. Finally, 14 full-text articles were accessed for eligibility criteria. Based on the pre-defined criteria and after critical appraisal (one article [29] was excluded) and 13 articles were included in the final analysis (Fig 1).

### Characteristics of included studies

A total of thirteen articles met the inclusion criteria. All the included studies were published between 2013 and 2019. We included both cross-sectional and case-control studies using an estimated sample size range from 284 [17] up to 528 [20]. About 5,108 women aged from 18–85 years were included to estimate the pooled prevalence of precancerous lesions of the cervix and its associated factors among women in Ethiopia Table 1.

**Table 1:** Descriptive summary of 13 studies included in the meta-analysis of the prevalence and determinants of the precancerous lesion of the cervix among Ethiopian women 2019.

Author, Year, Region	Study design and participants	Sample size	Prevalence	Associated Factors
Ameya et al. 2017 [13] SNNP	Retrospective cross sectional	513	9.9%	Old age
	Among Women above 17 yrs.			
Belayneh et al. 2019 [17] Amhara	Cross sectional among HIV positive women	284	9.9%	Age greater than 30 years old (AOR: 0.270; 95% CI: 0.076, 0.973), single in marital status (AOR: 4.901; 95% CI: 1.246–10.284), being commercial sex worker (AOR = 4.984; 95% CI: 2.15–9.965), had any other sexually transmitted infection (AOR = 4.515; 95% CI: 1.496–13.602), more than one sexual partner (AOR: 0.112; 95% CI: 0.029, 0.478), more than two children (AOR: 0.208; 95% CI: 0.060, 0.704) and with vaginal wall abnormality (AOR = 4.242; 95% CI: 1.423–12.676)
Deksissa et al. 2015 [11] Oromia	Cross sectional among women among clients screened at the Family Guidance Association of Ethiopia	334	12.9%	Early initiation of sexual intercourse (AOR [95% CI] 2.2 [1.1, 4.3])
Derbie et al. 2019 [18] Amhara	Cross sectional among women visiting the gynecology department of the Felege Hiwot Referral Hospital (FHRH)	428	14.3%	Being government employees (AOR: 0.24, 95% CI (0.07–0.85), p value 0.03).
Gedefaw et al. 2013 [10] SNNP	Cross sectional among HIV infected women in southern Ethiopia.	448	22.1%	Being currently on highly active antiretroviral treatment (AOR = 0.52, 95%CI: 0.35, 0.92), history of sexually transmitted disease (AOR = 2.30, 95%CI: 1.23, 4.29) and multiple sexual partner (AOR = 0.33, 95%CI: 0.20, 0.56)
Getinet et al. 2015 [16] Amhara	Comparative cross sectional	194 HIV+ 194 and HIV-	14.1%	Being HIV+ women (COR 1.9,95%CI:1.1 − 3.4, p = 0.036), Multiple sexual partnership (AOR 3.2, 95% CI: 1.1 − 10.0, p = 0.04), early ages of first sexual contact (<15 years) (AOR 5.2, 95% CI: 1.5 − 17.9, p = 0.009), parity greater than three (AOR 10.9, 95% CI: 4.2 − 16.8, p < 0.001) and long term oral contraceptive pills (OCP) use (AOR 11.9, 95% CI: 2.1 − 16.7, p = 0.02)
	Among women among HIV+ and HIV- women			
Kassa et al. 2019 [19] Amhara	Cross sectional among HIV+ women	435	20.2%	Having more than one lifetime sexual partner (AOR = 2.91, 95%CI:1.13, 7.52), a history of sexually transmitted disease (AOR = 4.04, 95%CI: 2.19, 7.44), age at first birth less than 18 years (AOR = 3.36, 95%CI: 1.79, 5.01) and baseline CD4 count less than 200 cells/mm3 (AOR = 7.51,95%CI: 3.58, 15.68)
Misgina et al. 2017 [14] Tigray	Cross sectional among women working in Almeda textile factory	342	6.7%	Being infected with sexually transmitted infections [AOR = 49.88, 95% CI: (16.59, 149.91)]
Teame et al. 2018 [16] Addis Ababa	Case—control	343 (114 cases and 229 controls)	12.8%	Women in the age group of40–49 years (AOR = 2.40, 95% CI (1.27–4.54)), history of STD (AOR = 3.20, 95% CI (1.26–8.10)), Multiple sexual partner (AOR = 2.17, 95% CI (1.01–4.67)), Multiple sexual partner of husband ((AOR = 3.03, 95% CI (1.25–7.33))
Teka et al. 2019 [20] SNNP	Cross sectional	528	27.7%	Having primary educational status (AOR [95% CI]) = 0.2 [0.1, 0.96)] and secondary educational status (AOR [95% CI]) = 0.1 [0.02,0.3]), having a history of smoking [AOR (95% CI) = 3.7 (1.4–9.9)], having two and more than two lifetime sexual partners [AOR (95% CI) = 2.2 (1.1–4.7)], having age at first sexual intercourse less than eighteen years [AOR (95% CI) = 6.6 (3.14–13.0)]
Temesgen et al. 2019 [21] Amhara	Cross sectional among clients (30-49yrs)	422	6.9%	Age group of 46–50 (AOR = 2.30, 95% CI (1.34–4.50)), multiparous ((≥4) (AOR = 2.16, 95% CI (1.36–5.89)), Starting sex before 20 years (AOR = 2.5, 95% CI (1.65–4.12)), Having two or more lifetime sexual partners (AOR = 3.86, 95% CI (2.37–6.69)), being HIV positive (AOR = 1.99, 95% CI (0.44–2.25)), history of HPV infection (AOR = 2.5, 95% CI (0.75–4.95)), history of sexually transmitted infections (AOR = 3.43, 95% CI (1.65–5.35)), smoke cigarettes (AOR = 2.01, 95% CI (0.78–4.11)), history of abortion (AOR = 1.55, 95% CI (0.88–3.21)), family history of cervical cancer (AOR = 1.85, 95% CI (0.77–3.99)),
Tesfalid et al. 2018 [23] unpublished paper SNNP	Case control among women aged 21–49 years who had undergone screening for precancerous cervical lesion by VIA.	295 (98 cases and 197 controls)		Women aged 30–39 years (AOR = 2.51, 95% CI: 1.03–6.08), monthly income < 42 USD and 43–66 USD (AOR = 3.41, 95% CI: 1.34–6.08; AOR = 3.63, 95% CI: 1.31–9.88), initiation of first sexual intercourse at age less than or equal to 20 (AOR = 2.39, 95% CI: 1.14–5.47), having more than one lifetime sexual partners (AOR = 4.70, 95% CI: 2.02–10.95), having a partner/ husband with more than one lifetime sexual partners (AOR = 2.98, 95% CI: 1.35–6.65)
Gessese et al. 2015 [22] Tigray	Case-control among HIV- positive women	348 (116 cases and 232 HIV-positive control)		HIV positive women with CD4 counts less than 350/mm3 (AOR = 1.6; 95% CI: 0.97, 2.68), married women (AOR = 2.3; 95% of CI: 1.28, 4.26), Women with two (AOR = 3.6; 95% CI: 1.7, 7.7), and three (AOR = 2.5; 95% CI: 1.2, 5.4) sexual partners

### Pooled prevalence of precancerous lesion of the cervix (meta-analysis)

**Fig 2:**
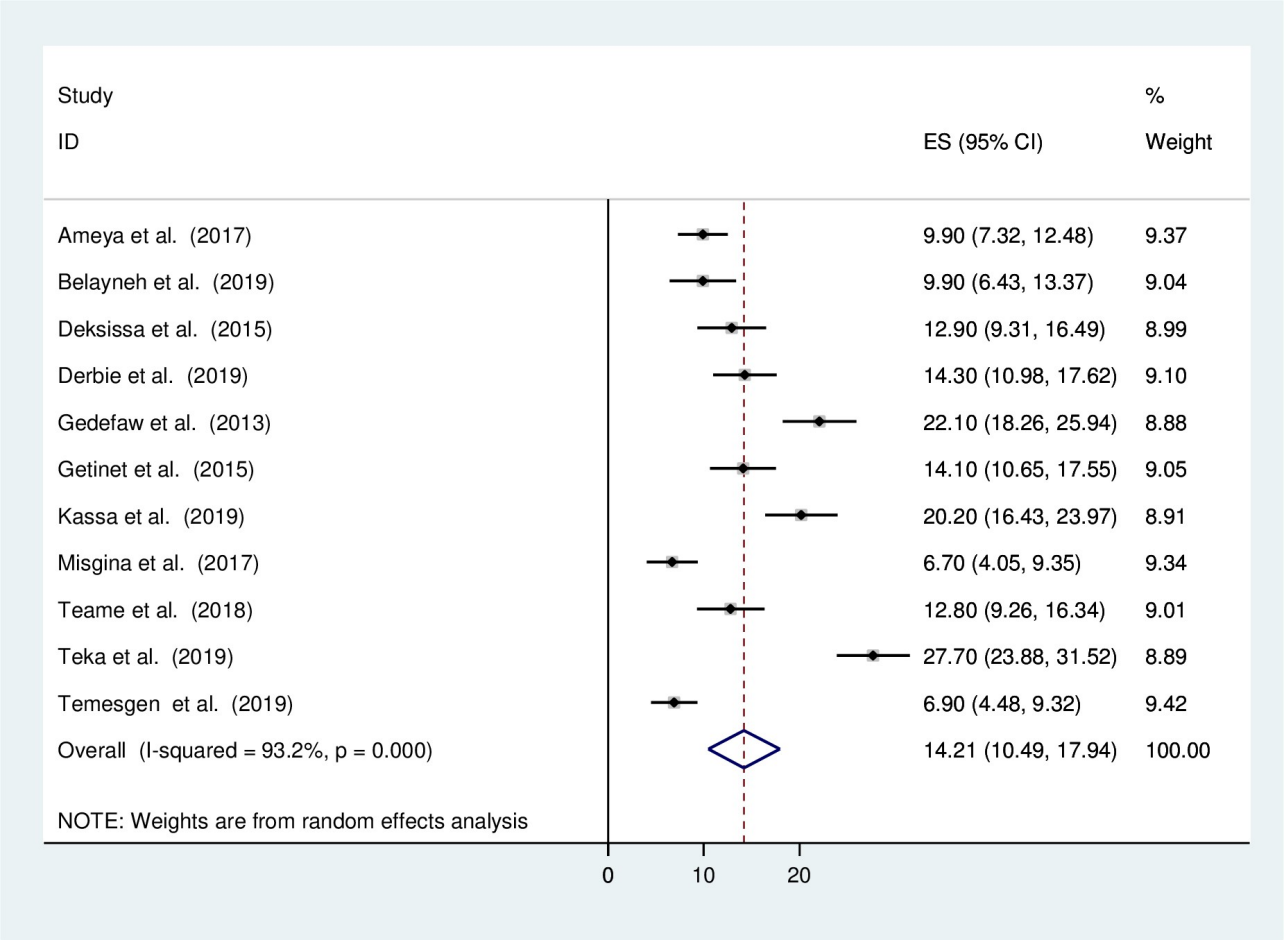
Forest plot of pooled prevalence of the precancerous lesions of the cervix among Ethiopian women. The diamond represents the pooled prevalence where us the outer edge of the diamond represents the 95% CI. The box with the back line represents the prevalence of each study with 95% CI.

The minimum prevalence of precancerous lesions of the cervix was 6.7% as reported in a study conducted in Tigray region [14]. The highest, 27.7% was observed in a study conducted in SNNPE [20]. The I^2^ test result showed high heterogeneity (I2, 93.2%, p = <0.001). Using the random effect analysis, the pooled prevalence of the precancerous lesions of the cervix among women in Ethiopia was 14.21% (95% CI (10.49, 17.94)) (Fig 2). After adjusting of publication bias using trim-and-fill method, the pooled prevalence was 9.43% (95% CI (5.23, 13.62).

### Subgroup analysis by study area

**Fig 3:**
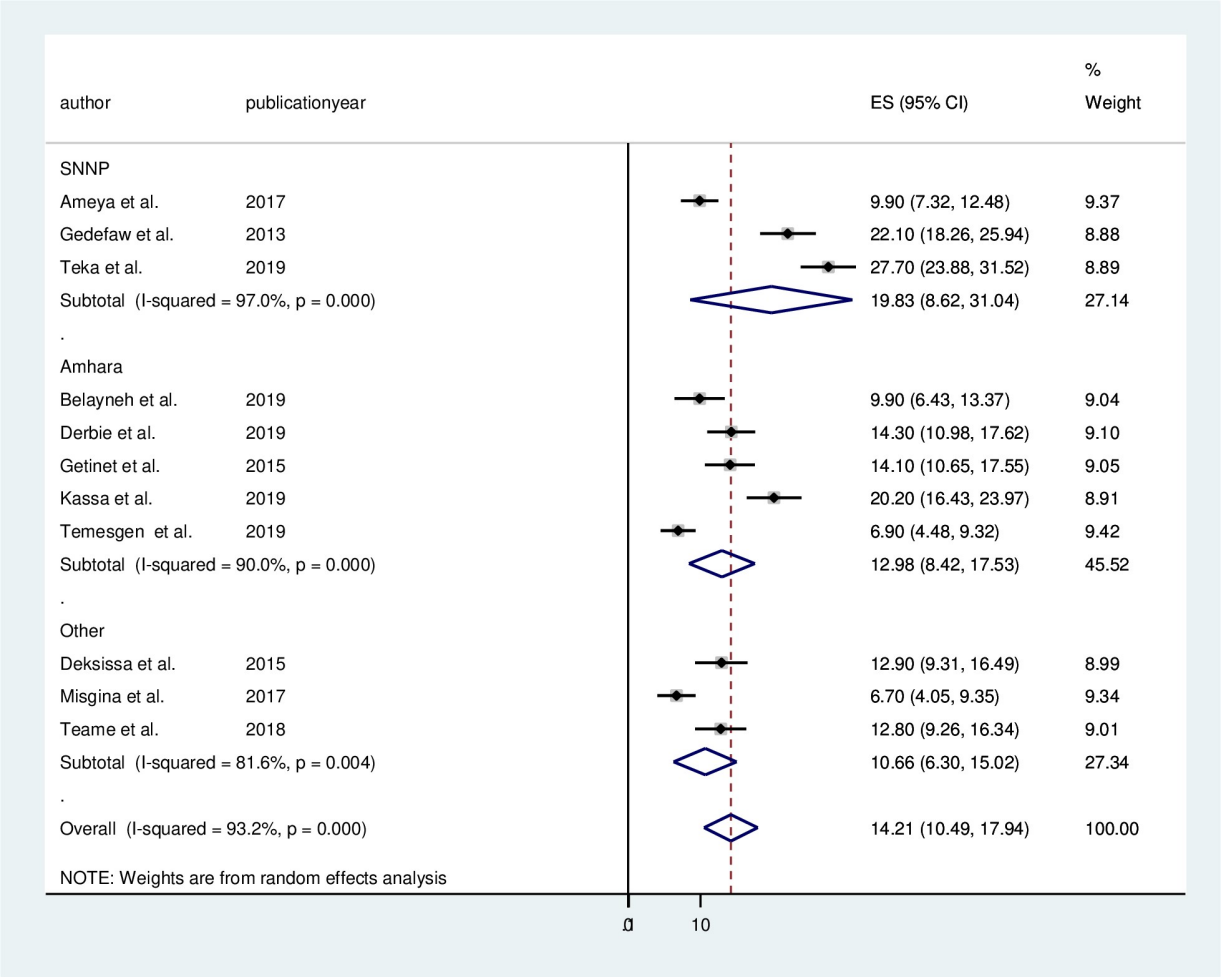
Sub-group analysis of the pooled prevalence of precancerous lesions of the cervix among Ethiopian women.

Subgroup analyses by study area were conducted to assess the potential heterogeneity between studies. Of the 11 studies, the highest prevalence of the precancerous lesion of the cervix occurred in studies conducted in SNNPE region (19.83%, 95% CI 8.62% to31.04%, I^2^ = 97.0%), followed by studies conducted in Amhara region (12.98%, 95% CI 8.42% to 17.53%, I^2^ = 90.0%). We summarize subgroup analyses by regions in (Fig 3).

### Publication bias

**Fig 4:**
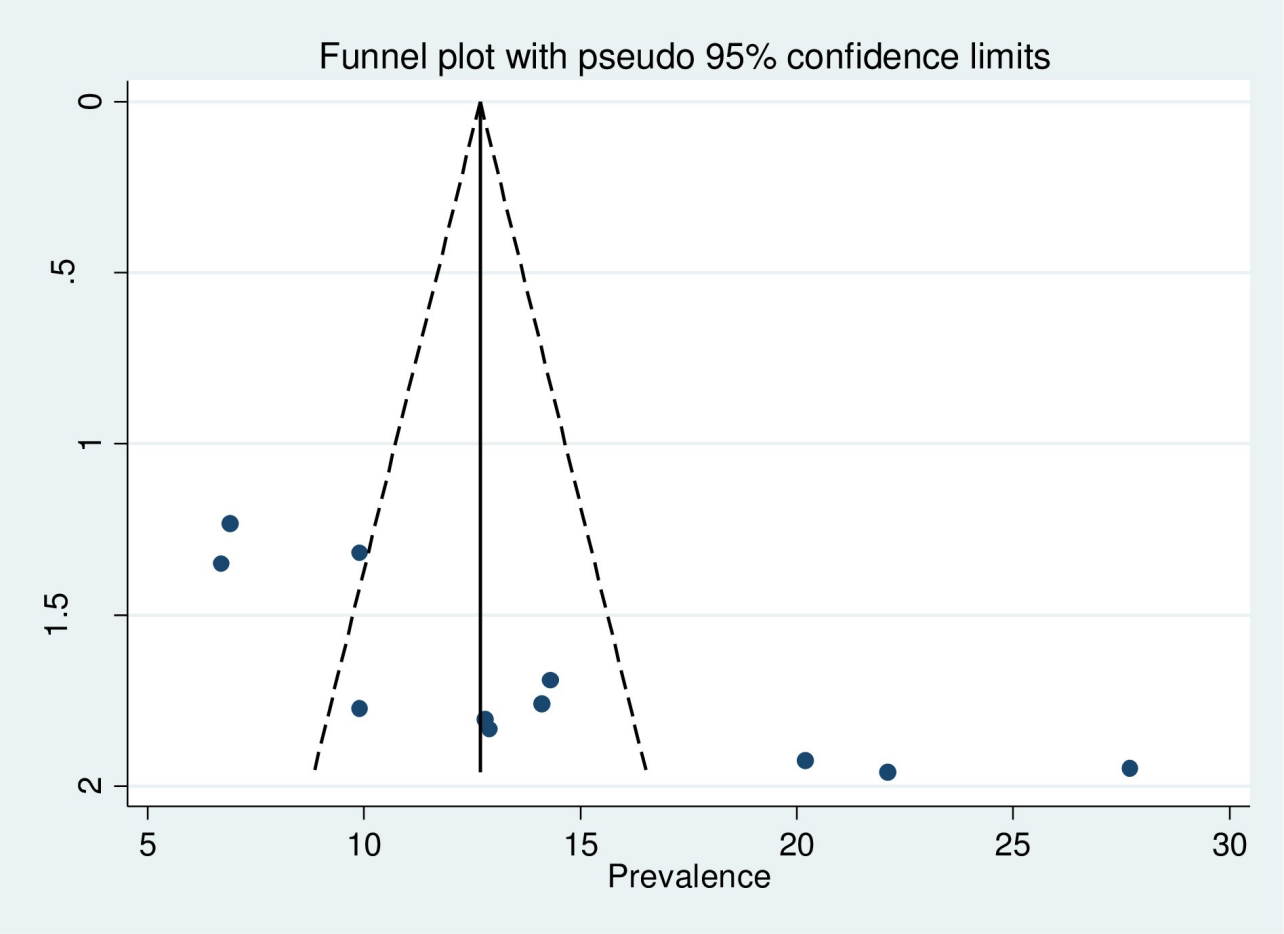
Funnel plot to assess publication bias for prevalence of precancerous lesion of the cervix in Ethiopia.

**Fig 5:**
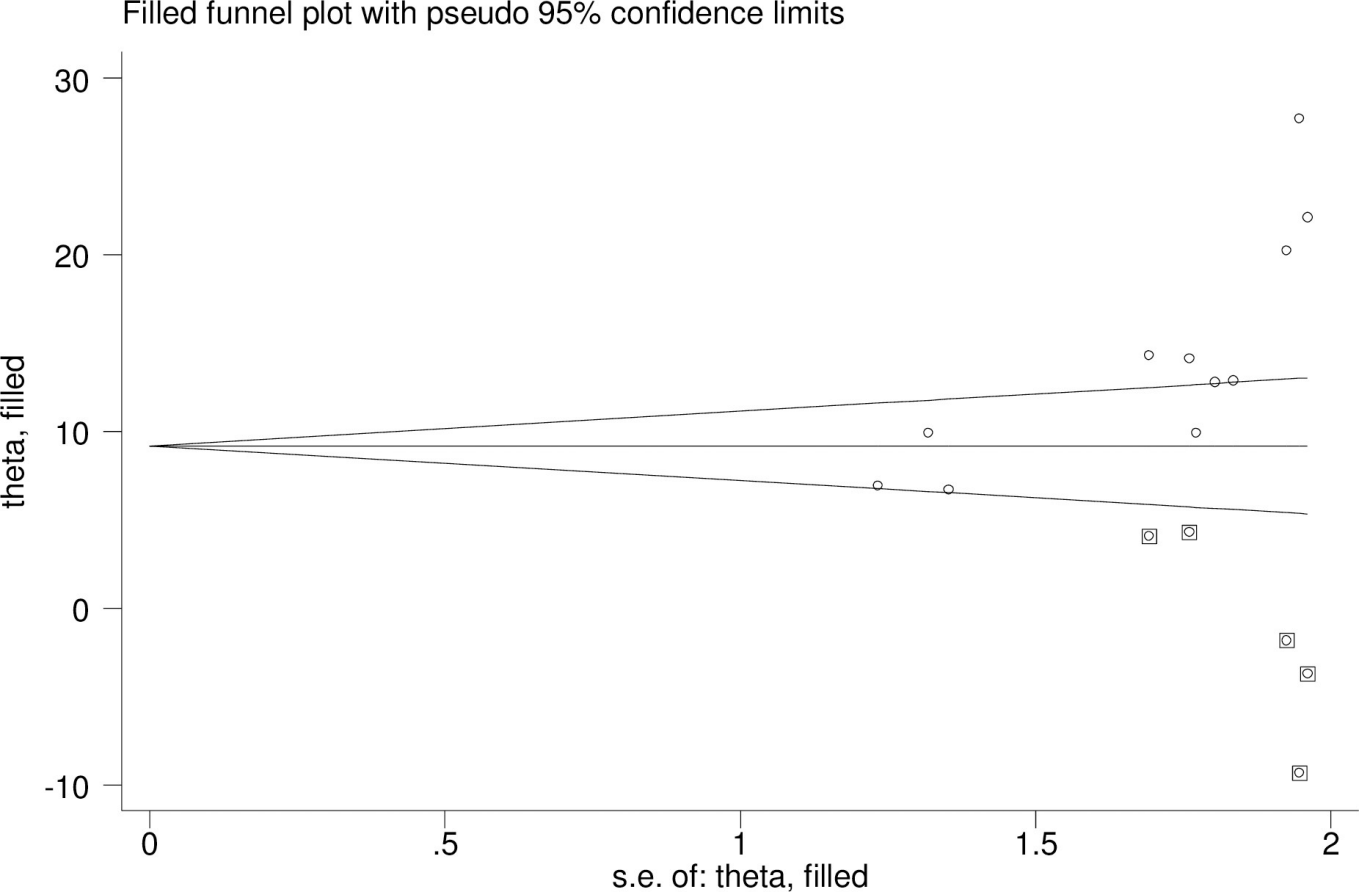
Funnel plot to show trim and fill analysis.

Evidence of publication bias was assessed using both Egger’s, and Begg’s tests with a p-value of less than 0.05 as a cut-off point and funnel plot. The funnel plot appeared asymmetrical and showed publication bias subjectively (Fig 4). The Egger weighted regression (P = 0.002) and Begg rank correlation test (p = 0.005) objectively revealed evidence of publication bias. To reduce this publication bias Trim and fill analysis was conducted and the result was depicted on (Fig 5).

### Sensitivity analysis

**Fig 6:**
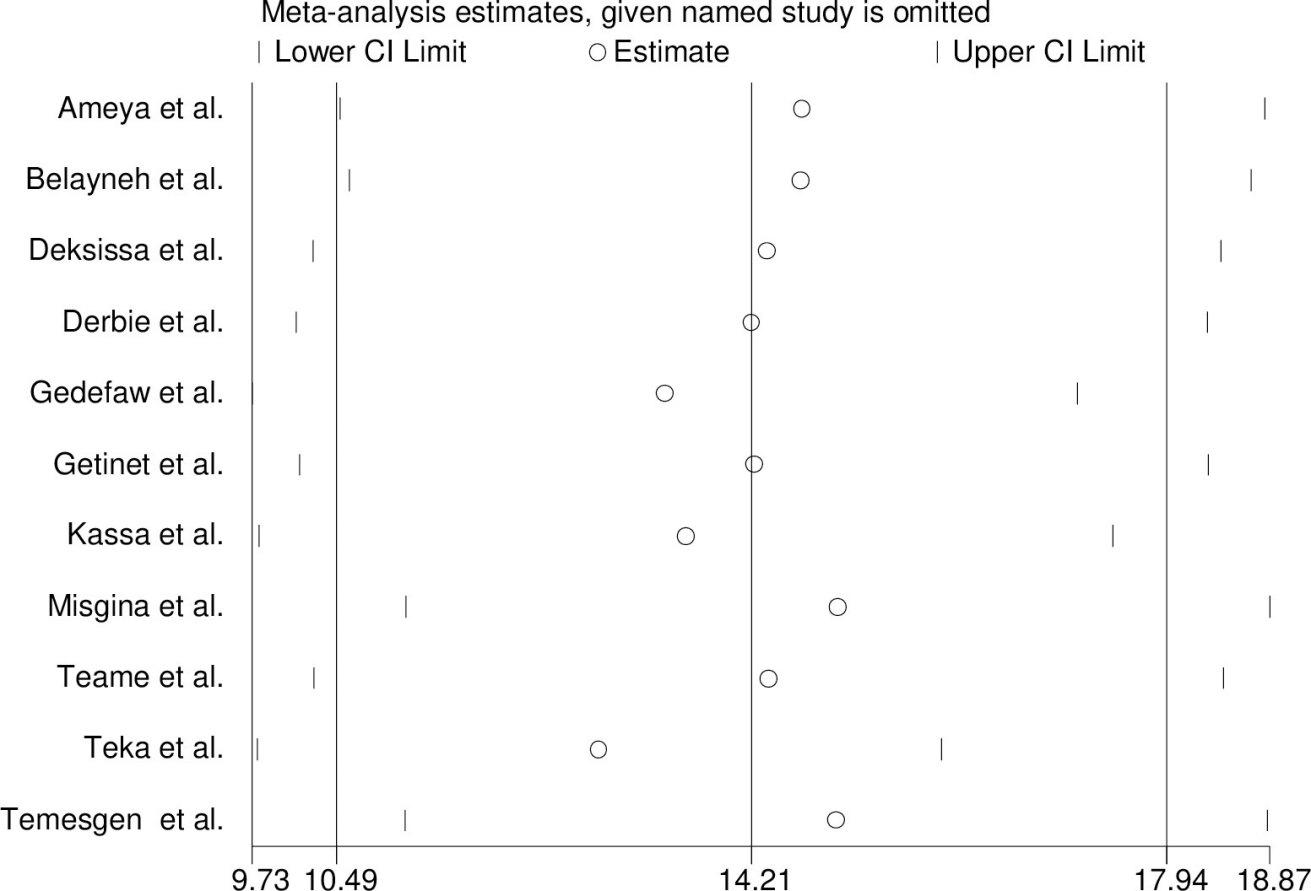
Sensitivity analysis.

The influence of a single study on the overall meta-analysis estimate was employed. Based on this the point estimate of its omitted analysis lies within the confidence interval of the combined analysis (Fig 6). This suggested that no single study unduly influenced the overall prevalence estimate of a precancerous lesion of the cervix among Ethiopian women.

### Determinants of the precancerous lesions of the cervix among Ethiopian women

#### Early initiation of first sexual intercourse and the precancerous lesions of the cervix

**Fig 7:**
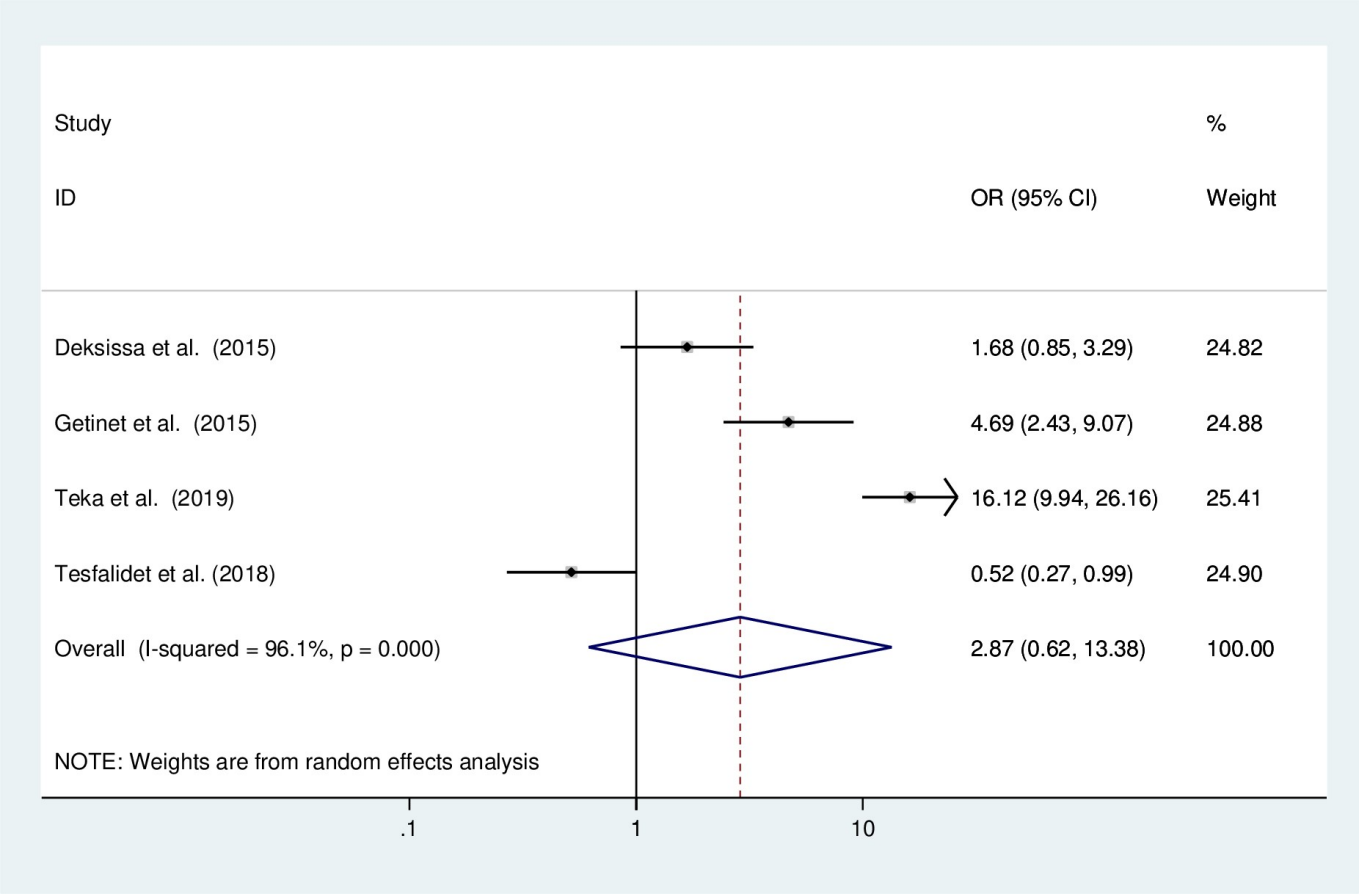
The pooled odds ratio of the association of early initiation of first sexual intercourse and the precancerous lesions of the cervix among Ethiopian women.

In this meta-analysis, we examined the association between early initiation of first sexual intercourse and precancerous lesions of the cervix depending on four studies [11, 12, 20, 23]. The result of the test statistics showed severe heterogeneity (I^2^ = 96.1% and p<0.0001). Therefore, a random effect meta-analysis model was employed to determine the association (Fig 7). Based on this, the occurrence of precancerous lesions of the cervix was not significantly associated with early initiation of first sexual intercourse.

#### The association of multiple sexual partners and the occurrence of precancerous lesions of the cervix

**Fig 8:**
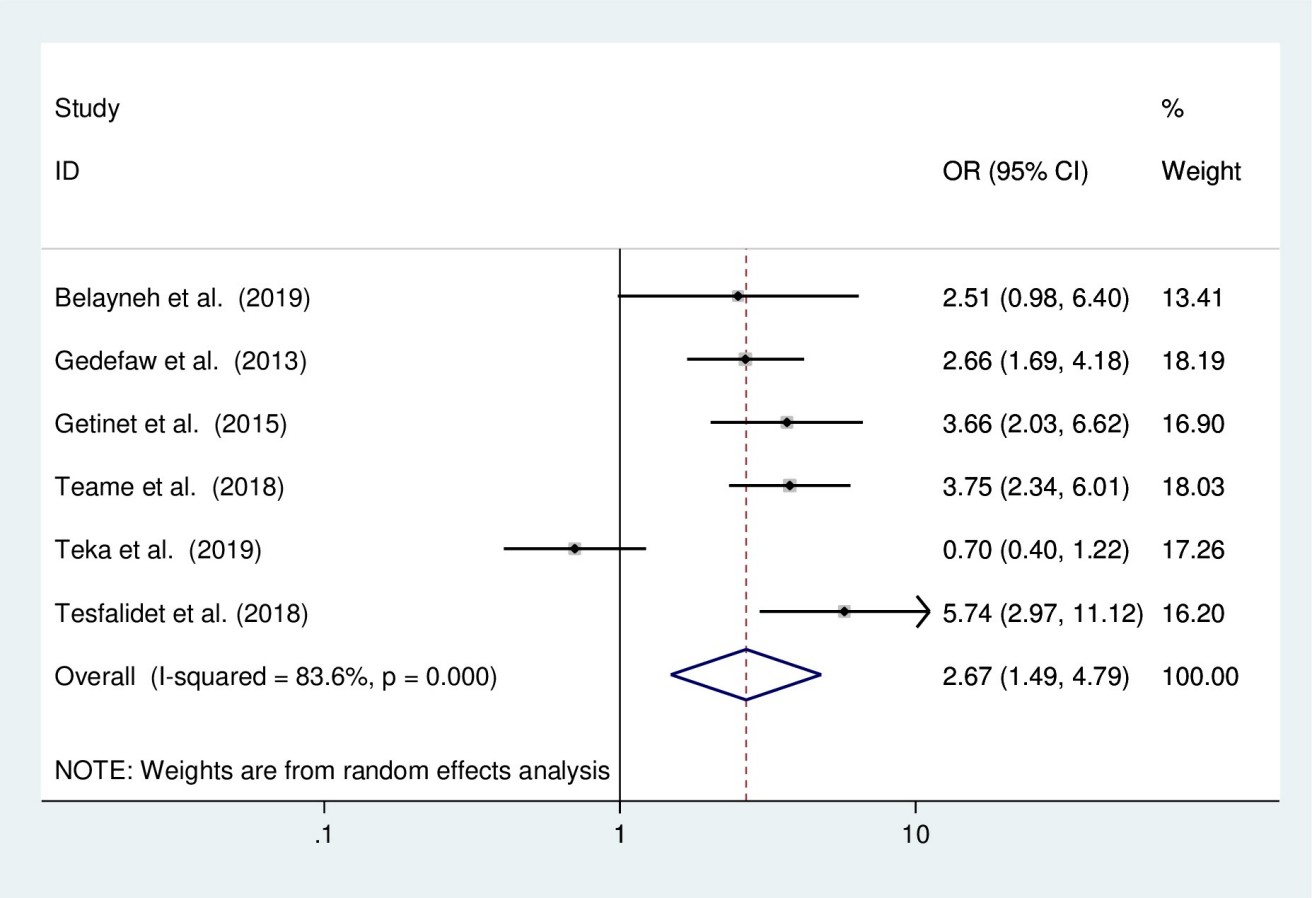
The pooled odds ratio of the association of having multiple sexual partners and the precancerous lesion of the cervix among Ethiopia women.

A meta-analysis of six studies [10, 12, 16, 17, 20, 23] was conducted to determine the association between having multiple sexual partners and the occurrence of the precancerous lesions of the cervix. The result of the test statistics indicated the presence of severe heterogeneity (I^2^ = 83.6% and p<0.0001). Therefore, a random effect meta-analysis model was employed to determine the association (Fig 8). The results from these studies revealed that the occurrence of the precancerous lesions of the cervix was significantly associated with the presence of multiple sexual partners. Based on these women who had multiple sexual partners in their lifetime were 2.67 times more likely to have the precancerous lesions of the cervix than those who had not multiple sexual partners (OR:2.67 CI: 1.49,4.79).

#### The association of history of STI and the precancerous lesion of the cervix

**Fig 9:**
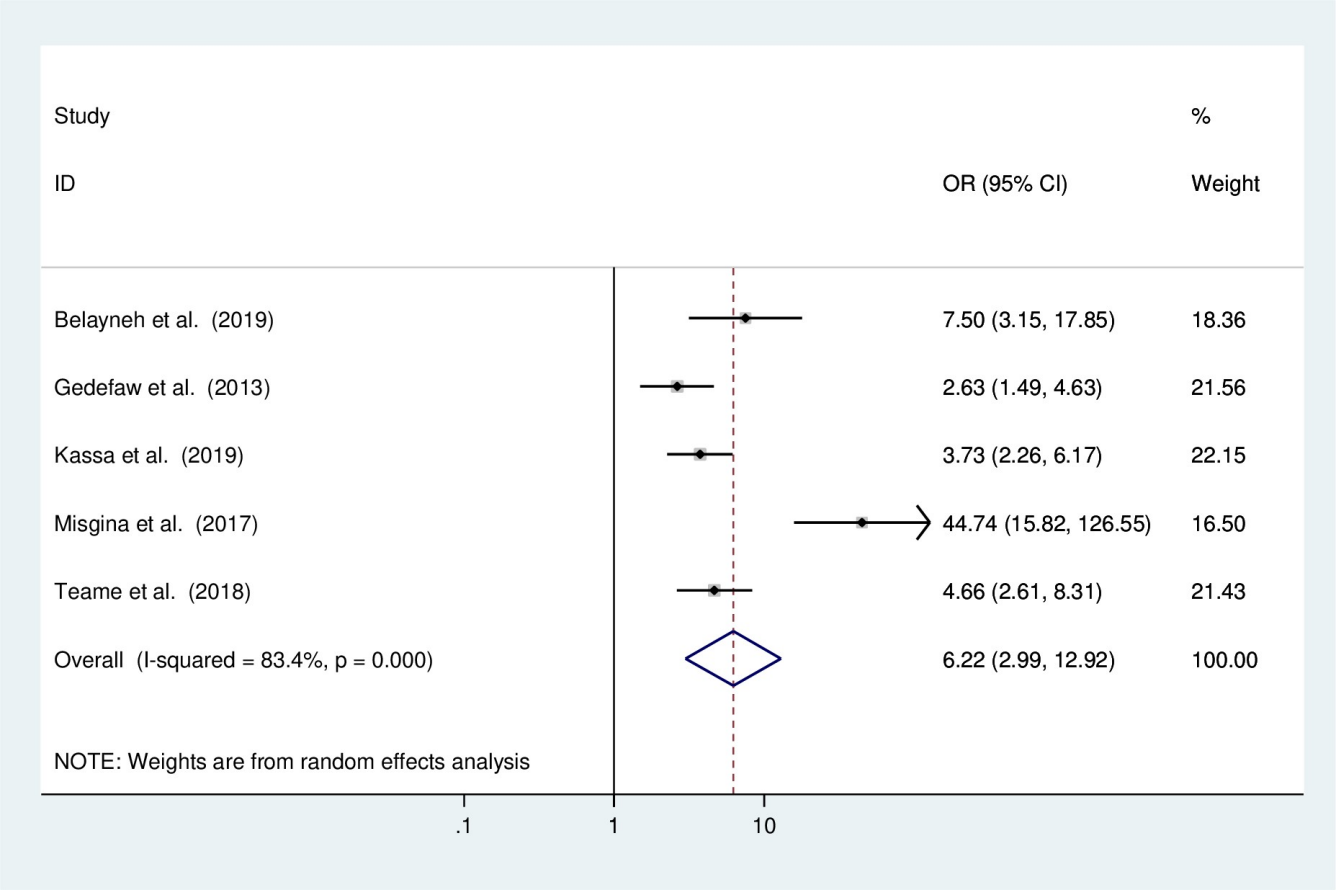
The pooled odds ratio of the association of multiple sexual partners and the precancerous lesions of the cervix among Ethiopian women.

The association between history of STI and the precancerous lesion of the cervix among Ethiopian women was analysed based on five studies [10, 14, 16, 17, 19]. The result of the test statistics indicated the presence of severe heterogeneity (I^2^ = 83.4% and p<0.0001). Therefore, a random effect meta-analysis model was employed to determine the association (Fig 9). The results from these studies revealed that the occurrence of precancerous lesions of the cervix was significantly associated with a history of STI. Accordingly, women who had history of STI were 6.22 times more likely to have the precancerous lesions of the cervix than those with no history of STI (OR:6.22 CI: 2.99,12.92).

## Discussion

This review was conducted to determine the pooled prevalence and determinants of the precancerous lesions of the cervix among Ethiopian women using published and unpublished studies. Understanding the prevalence and determinants of the precancerous lesions of the cervix among women is a base for the establishment of screening and vaccination programs. This indirectly could decrease the morbidity and mortality caused by cervical cancer.

The pooled meta-analysis of this review found that the prevalence of precancerous lesions of the cervix among Ethiopian women was 14.21% (95% CI (10.49, 17.94)). After adjusting of publication bias using trim-and-fill method, the pooled prevalence was 9.43% (95% CI (5.23, 13.62). This is lower than the previous studies conducted in Thailand (13.3%) [30], Uganda (13.6%) [31], Botswana (15.2%) [32], Sudan (16%) [33], Nigeria (16%) [34], Bangladesh (18%) [33], Kenya (26.7%) [35], Greece (49.5%) [36], Zambia (76%) [37]. It is also lower than the pooled prevalence of precancerous lesions of the cervix among HIV-positive women in sub-Saharan Africa 25.6% [38]. On the other hand, it is higher than the reports of Cameron (3.9%) [39], Rwanda (5.9%) [40], Uganda (7.8%) [41], and comparable with study in Tanzania (9.7%) [24], Madagascar (11.3) [41], Malawi (12.4%) [41], and Latin America (12%) [42].

There could be several reasons for the difference in the prevalence of the precancerous lesions of the cervix from one country to another. These may include the existence and consistency of screening programs and management options implemented in these countries; differences in the sexual practices of the women studied, and the differences in the study populations.

Subgroup analysis of studies based on region revealed that the highest prevalence of precancerous lesions of the cervix occurred in SNNPE region (19.83%), followed by studies conducted in the Amhara region (12.98%). Such regional difference may be due to differences in the study population, culture, religion and sociodemographic factors.

In this review, we have identified the pooled determinants of precancerous lesions of the cervix among Ethiopian women. The occurrence of precancerous lesions of the cervix was significantly associated with the presence of multiple sexual partners. Women who had multiple sexual partners in their lifetime were 2.67 times more likely to have the precancerous lesion of the cervix than those who had no multiple sexual partners. This is in line with studies done in Côte D’Ivoire [43], Swaziland [44], Kenya [45], united states [46], Italy [47], and Tanzania [48]. Women having multiple sexual partners may be exposed to factors predisposing to the precancerous lesions of the cervix such as HIV and STI.

Other determinant of precancerous lesions of the cervix in our review was a history of STI. The results from these studies revealed that the occurrence of the precancerous lesions of the cervix was significantly associated with a history of an STI. Accordingly, women who had a history of STI were 6.22 times more likely to have the precancerous lesions of the cervix than those with no history of an STI. This is supported by research done in Tanzania [48], Kenya [45, 49], Swaziland [44], Côte D'Ivoire [50], Zimbabwe [51], and India [52].

This may be due to the fact that infection with an STI in the presence of HPV increased the risk of CIN by causing inflammation which facilitates HPV persistence and hence cervical lesion and carcinogenesis [53]. HPV is mainly transmitted through sexual contact and most people are infected with HPV shortly after the onset of sexual activity. Cervical cancer is caused by sexually acquired infection with certain types of HPV. Two HPV types (16 and 18) cause 70% of cervical cancers and pre-cancerous cervical lesions [54].

## Conclusion

The pooled prevalence of the precancerous lesions of the cervix among Ethiopian women was 14.21% and after adjusting publication bias by After adjusting of publication bias using trim-and-fill method, the pooled prevalence was 9.43%. It was associated with having multiple sexual partners and history of sexually transmitted infections.

## Supporting information

S1 Table PRISMA checklist: Recommended items addressed in our systematic review and meta-analysis. (PDF)

S2 Table Quality assessment of included studies using the STROBE checklist. (PDF)

S1 File Search strategy conducted between the 1st of October to the 1st of November, 2019. (PDF)

## List of abbreviations

CIN: Cervical Intraepithelial Neoplasia
HPV: Human Papillomavirus
JBI: Joanna Briggs Institute
PRISMA: Preferred Reporting Items for Systematic Reviews and Meta-Analysis
SNNPE: Southern Nations, Nationalities and Peoples of Ethiopia
STI: Sexually Transmitted Infection
VIA: Visual Inspection of the cervix with Acetic acid
VILI: Visual Inspection with Lugol’s Iodine
