# Supplementary material for: Precancerous lesions of the cervix and its determinants among Ethiopian women: Systematic review and meta-analysis
Source: PLoS One. 2020 Oct 28;15(10):e0240353. doi: 10.1371/journal.pone.0240353 (PMC7592780; doi:10.1371/journal.pone.0240353)
Supplement: S2 Table — (PDF) [file pone.0240353.s002.pdf]

**S2_Table. Quality assessment of included studies using the STROBE checklist**

| | Study | Score (out of 22) | Score (percentage) | Quality |
|---|---|---|---|---|
| 1 | Ameya et al.2017 | 18.5 | 84.1% | High |
| 2 | Belayneh et al. 2019 | 19 | 86.4% | High |
| 3 | Deksissa et al. 2015 | 20 | 90.9% | High |
| 4 | Derbie et al. 2019 | 21 | 95.5% | High |
| 5 | Gedefaw et al. 2013 | 20 | 90.9% | High |
| 6 | Getinet et al. 2015 | 21 | 95.5% | High |
| 7 | Kassa et al. 2019 | 20 | 90.9% | High |
| 8 | Misgina et al. 2017 | 17 | 77.3% | High |
| 9 | Teame et al. 2018 | 20 | 90.9% | High |
| 10 | Teka et al. 2019 | 19 | 86.4% | High |
| 11 | Temesgen et al. 2019 | 17 | 77.3% | High |
| 12 | Tesfalid et al.2018 | 16 | 72.7% | Low |
| 13 | Gessese et al. 2015 | 16 | 72.7% | Low |

STROBE: Strengthening the Reporting of Observational Studies in Epidemiology

STROBE Statement—The Strengthening the Reporting of Observational Studies in Epidemiology (STROBE) Statement: Checklist of Items That Should Be Addressed in Reports of Observational Studies

| | Item No | Recommendation |
|---|---|---|
| **Title and abstract** | 1 | (*a*) Indicate the study's design with a commonly used term in the title or the abstract |
| | | (*b*) Provide in the abstract an informative and balanced summary of what was done and what was found |
| **Introduction** | | |
| Background/rationale | 2 | Explain the scientific background and rationale for the investigation being reported |
| Objectives | 3 | State specific objectives, including any prespecified hypotheses |
| **Methods** | | |
| Study design | 4 | Present key elements of study design early in the paper |
| Setting | 5 | Describe the setting, locations, and relevant dates, including periods of recruitment, exposure, follow-up, and data collection |
| Participants | 6 | (a)Case–control study: Give the eligibility criteria, and the sources and methods of case ascertainment and control selection. Give the rationale for the choice of cases and controls.<br>Cross-sectional study: Give the eligibility criteria, and the sources and methods of selection of participants.<br>(b) Case–control study: For matched studies, give matching criteria and the number of controls per case. |
| Variables | 7 | Clearly define all outcomes, exposures, predictors, potential confounders, and effect modifiers. Give diagnostic criteria, if applicable |
| Data sources/ measurement | 8* | For each variable of interest, give sources of data and details of methods of assessment (measurement). Describe comparability of assessment methods if there is more than one group |
| Bias | 9 | Describe any efforts to address potential sources of bias |
| Study size | 10 | Explain how the study size was arrived at |
| Quantitative variables | 11 | Explain how quantitative variables were handled in the analyses. If applicable, describe which groupings were chosen and why |
| Statistical methods | 12 | (*a*) Describe all statistical methods, including those used to control for confounding |
| | | (*b*) Describe any methods used to examine subgroups and interactions |
| | | (*c*) Explain how missing data were addressed |
| | | (*d*) Case–control study: If applicable, explain how matching of cases and controls was addressed.<br>Cross-sectional study: If applicable, describe analytical methods taking account of sampling strategy. |
| | | (*e*) Describe any sensitivity analyses |
| **Results** | | |

| | | |
|---|---|---|
| Participants | 13* | (a) Report numbers of individuals at each stage of study—eg numbers potentially eligible, examined for eligibility, confirmed eligible, included in the study, completing follow-up, and analyzed |
| | | (b) Give reasons for non-participation at each stage |
| | | (c) Consider use of a flow diagram |
| Descriptive data | 14* | (a) Give characteristics of study participants (eg demographic, clinical, social) and information on exposures and potential confounders |
| | | (b) Indicate number of participants with missing data for each variable of interest |
| Outcome data | 15* | Case–control study: Report numbers in each exposure category or summary measures of exposure.<br><br>Cross-sectional study: Report numbers of outcome events or summary measures. |
| Main results | 16 | (*a*) Give unadjusted estimates and, if applicable, confounder-adjusted estimates and their precision (eg, 95% confidence interval). Make clear which confounders were adjusted for and why they were included |
| | | (*b*) Report category boundaries when continuous variables were categorized |
| | | (*c*) If relevant, consider translating estimates of relative risk into absolute risk for a meaningful time period |
| Other analyses | 17 | Report other analyses done—eg analyses of subgroups and interactions, and sensitivity analyses |
| **Discussion** | | |
| Key results | 18 | Summarize key results with reference to study objectives |
| Limitations | 19 | Discuss limitations of the study, taking into account sources of potential bias or imprecision. Discuss both direction and magnitude of any potential bias |
| Interpretation | 20 | Give a cautious overall interpretation of results considering objectives, limitations, multiplicity of analyses, results from similar studies, and other relevant evidence |
| Generalizability | 21 | Discuss the generalizability (external validity) of the study results |
| **Other information** | | |
| Funding | 22 | Give the source of funding and the role of the funders for the present study and, if applicable, for the original study on which the present article is based |
