# Supplementary material for: Precancerous lesions of the cervix and its determinants among Ethiopian women: Systematic review and meta-analysis
Source: PLoS One. 2020 Oct 28;15(10):e0240353. doi: 10.1371/journal.pone.0240353 (PMC7592780; doi:10.1371/journal.pone.0240353)
Supplement: S1 File — (PDF) [file pone.0240353.s003.pdf]

Search strategy conducted between the 1st of October to the 1st of November, 2019

1. (Precancerous AND lesion AND ("cervix uteri"[MeSH Terms] OR ("cervix" AND "uteri" OR "cervix uteri" OR "cervix" AND ("Ethiopia"[MeSH Terms]

2.(Determinants AND precancerous AND lesion AND ("cervix uteri"[MeSH Terms] OR ("cervix" AND "uteri") OR "cervix uteri" OR "cervix” AND ("Ethiopia"[MeSH Terms].

3.(Factors AND precancerous AND lesion AND ("cervix uteri"[MeSH Terms] OR ("cervix" AND "uteri" OR "cervix uteri" OR "cervix" AND ("Ethiopia"[MeSH Terms]

4.(("Acetic acid"[MeSH Terms] OR ("acetic" AND "acid") OR "acetic acid" OR "acetates"[MeSH Terms] OR "acetates" OR ("acetic" AND "acid" AND positive AND lesion AND ("cervix uteri"[MeSH Terms] OR ("cervix" AND "uteri") OR "cervix uteri" OR "cervix") AND ("Ethiopia"[MeSH Terms]

5. (Intraepithelial AND lesion AND ("cervix uteri"[MeSH Terms] OR ("cervix" AND "uteri") OR "cervix uteri" OR "cervix" AND ("HIV seropositivity"[MeSH Terms] OR ("HIV" AND "seropositivity" OR "HIV seropositivity" OR ("HIV" AND "positive") OR "HIV positive") AND ("women"[MeSH Terms] OR "women")) AND ("Ethiopia"[MeSH Terms] AND "Ethiopia".

6. VIA AND positive AND lesions AND ("cervix uteri"[MeSH Terms] OR ("cervix" AND "uteri") OR "cervix uteri" OR "cervix") AND ("Ethiopia"[MeSH Terms] AND "Ethiopia")

7. ("Precancerous conditions"[MeSH Terms] OR ("precancerous" AND "conditions" OR "precancerous conditions" OR "premalignant") AND lesion AND ("cervix uteri"[MeSH Terms] OR ("cervix" AND "uteri") OR "cervix uteri" OR "cervix") AND ("Ethiopia"[MeSH Terms] AND "Ethiopia").

Data base	Searching terms	Number of studies
Google scholar	Precancerous AND lesion AND ("cervix uteri" OR ("cervix" AND "uteri" OR "cervix uteri" OR "cervix" AND ("Ethiopia"<br><br>Determinants AND precancerous AND lesion AND ("cervix uteri" OR ("cervix" AND "uteri") OR "cervix uteri" OR "cervix” AND ("Ethiopia".<br><br>"Acetic acid" OR "acetic" AND "acid" OR "acetic acid" OR "acetates" OR "acetates" OR ("acetic" AND "acid" AND positive AND lesion AND ("cervix uteri" OR ("cervix" AND "uteri") OR "cervix uteri" OR "cervix") AND ("Ethiopia"	150
